# Respiration-coordinated attentional switch from feedforward to top-down informational flow directed by the basal forebrain: layer-specific blanket inhibition of pyramidal cells by neurogliaform cells in the piriform cortex

**DOI:** 10.3389/fncir.2026.1859031

**Published:** 2026-06-19

**Authors:** Kensaku Mori, Hitoshi Sakano

**Affiliations:** 1 RIKEN Center for Brain Science, Wako, Saitama, Japan; 2 Department of Animal Behaviors, School of Veterinary Medicine, University of Tokyo, Bunkyo-Ku, Tokyo, Japan

**Keywords:** attentional switch in the basal forebrain, blanket inhibition in the cortical layers, feedforward sensory signals, lateral olfactory stream, respiration-coordinated operational states, top-down cognitive signals

## Abstract

When we are awake, we pay attention to a specific object in the surrounding world. Feedforward transmission of sensory information is enhanced in the cortico-cortical networks during the inhalation phase of the respiratory cycle for identification and evaluation of sensory information. Memory engrams of the associated scene are recalled by the sensory signal as a search tag. In the mammalian olfactory system, a topographic pattern of activated glomeruli in the olfactory bulb is transferred to the piriform cortex via the anterior olfactory nucleus, and then to higher cognitive areas. The odor map is utilized as a QR code (two-dimensional bar code) for recollection of the associative memory scene. Higher cognitive areas generate imagery of the multisensory cognitive scene, determine the valence of input information, and make behavioral decisions during the exhalation phase of respiration. The cognitive scene information for valence decision is transmitted back to the olfactory cortical areas in the top-down direction through the same set of pyramidal cells as used for the feedforward transmission of sensory information. The top-down information not only directs the output behaviors and generates emotional states but also rewrites the existing memory engrams. Then, How is this attentional switch from the surrounding outer world to the cognitive inner world regulated in a respiration-phase correlated manner? We propose that the basal forebrain inputs to neurogliaform cells in the piriform cortex may play a key role in switching attention by layer-specific blanket inhibition of pyramidal cells.

## Introduction

During wakefulness, our attention is directed toward either the surrounding outside world or cognitive inside world. Changes in the environmental situation are detected by the sensory systems, then identified and evaluated in higher cognitive areas in reference to the associative memory scene. This feedforward transmission of sensory information to the cognitive areas is promoted in the inhalation phase of respiratory cycle. During this period, our attention is directed toward the outside world. Once the changes are identified and evaluated, the decision is then made for output behaviors. When the situation is preferable and satisfactory, we become motivated and happy by the release of positive neuromodulators, e.g., dopamine and oxytocin. When the situation is unfavorable and not pleasant, we become depressed and unhappy by the release of stress hormones, e.g., adrenocorticotropic hormone (ACTH). This top-down transduction of emotional and behavioral signals occurs in the exhalation phase of the respiratory cycle. During this period, our attention is directed toward the cognitive inner world. It is interesting to study at the neural-circuit level, how the attention is generated and switched in correlation to the respiratory cycle. This respiration-coordinated attentional switch can be intentionally regulated. For example, the inhalation phase is prolonged or repeated for further investigation, and the exhalation phase is extended for more consideration.

**FIGURE 1:**
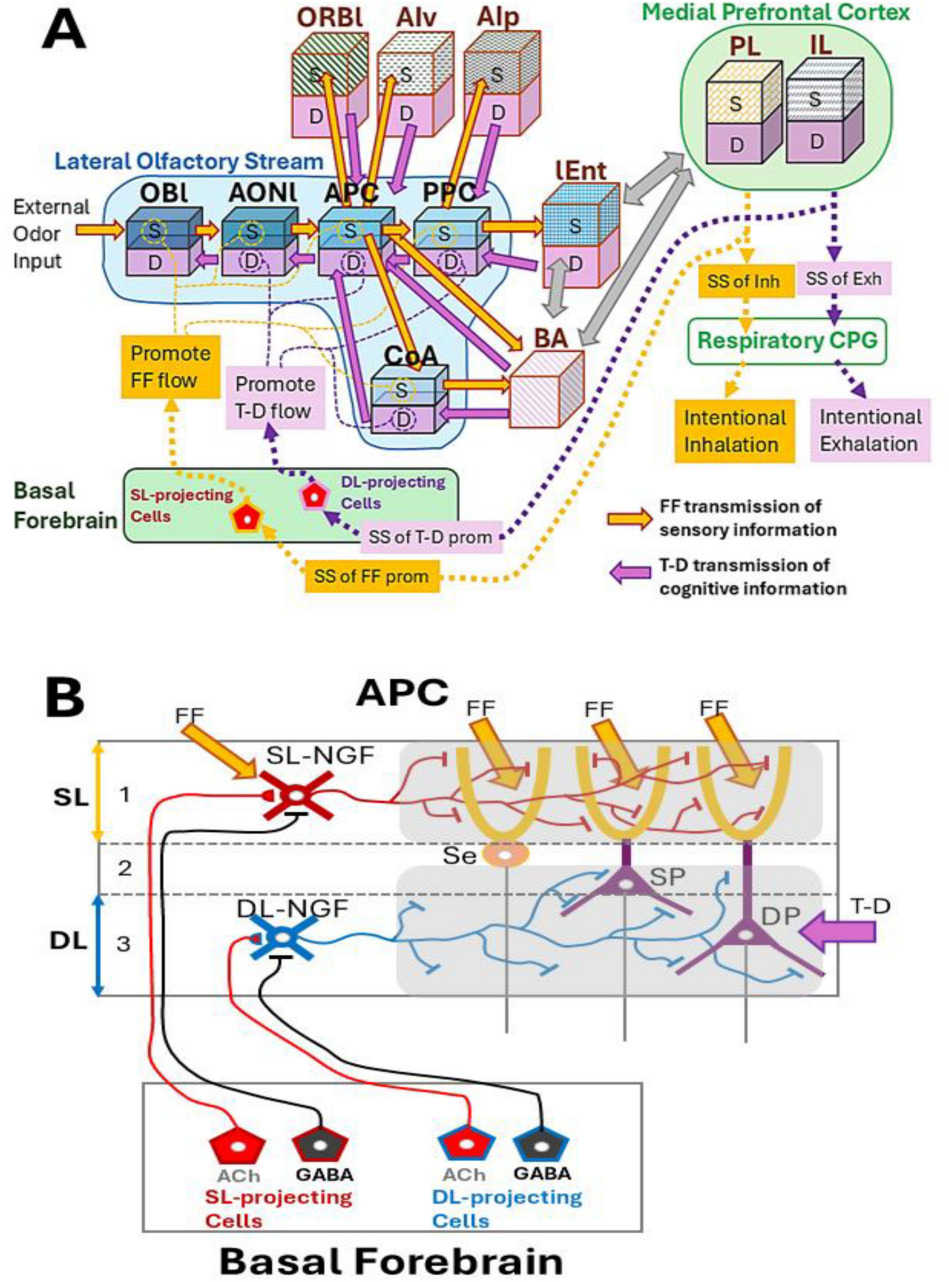
A neural network model of layer-specific input gating in the olfactory cortex. **(A)** Networks in the olfactory cortex and higher cognitive areas that mediate feedforward transmission of external odor information and top-down transmission of cognitive-scene signals. Orange arrows indicate feedforward (FF) transmission of odor information in the lateral olfactory stream (circled by a blue line), i.e., the lateral map of olfactory bulb (OBl) → lateral part of anterior olfactory nucleus (AONl) → anterior piriform cortex (APC) → posterior piriform cortex (PPC) and cortical nucleus of amygdala (CoA). Feedforward transmission occurs during the inhalation phase via synapses in the superficial layer (S) of these olfactory areas. External odor information is further transmitted to the higher multisensory cognitive areas (brown bold letters) that include the basal amygdala (BA), lateral entorhinal cortex (lEnt), lateral orbitofrontal cortex (ORBl), ventral agranular insular cortex (AIv), and posterior agranular insular cortex (AIp). In contrast, the higher cognitive areas send top-down (T-D) cognitive scene information (purple solid arrows) back to the olfactory areas during the late exhalation phase. Top-down transmission occurs via synapses in the deep layer (D) of the olfactory areas. We postulate that the medial prefrontal cortex, including the prelimbic cortex (PL) and infralimbic cortex (IL), coordinately generates start-signals of intentional inhalation (SS of Inh, orange dotted arrows to the respiratory central-pattern-generator, CPG) and activates a subset of basal forebrain cells that project axons to the superficial layer (S) of the olfactory areas. Activation of these basal forebrain cells may promote the feedforward transmission (orange dotted arrows to the basal forebrain). Such coordination may cause the facilitation of feedforward transmission of external odor information during intentional inhalation. We also postulate that the medial prefrontal cortex coordinately generates start-signals of intentional exhalation (SS of Exh, purple dotted arrows to the respiratory CPG) and activates a different subset of basal forebrain cells that project axons to the deep layer (DL). Activation of the basal forebrain cells may promote the T-D transmission (purple dotted arrows to the basal forebrain). Such a network mechanism may cause the facilitation of T-D transmission of cognitive scene information during intentional exhalation which typically occurs during the late exhalation phase. In the higher cognitive areas, layers 1∼3 are referred to as superficial layer (S), while layers 5 and 6 are as deep layer (D). **(B)** A model of basal forebrain (BF) projection to NGF cells in the anterior piriform cortex (APC). BF projection-neurons are classified into cholinergic neurons (red) and GABAergic neurons (black), both of which project their axons to NGF cells and inhibit them. NGF cells densely project their axons to a specific layer of each olfactory area and direct blanket inhibition of all dendrites of principal cells in the layer (shadowed in gray). We propose that a subset of BF neurons (SL-projecting cells) connects to NGF cells in the superficial layer (SL-NGF cells) and selectively inhibit them, disinhibiting the SL dendrites of principal cells. We also propose that a different subset of BF cells (DL-projecting BF cells) projects to NGF cells in the deep layer (DL-NGF cells) and selectively inhibits them, thus disinhibiting the DL dendrites of pyramidal cells. Orange arrows with FF indicate the feedforward odor inputs. A purple arrow with T-D shows the top-down cognitive-scene inputs. Se (semilunar cell), SP (superficial pyramidal cell), DP (deep pyramidal cell).

Based on the current-source-density analysis of local field potentials in the rat olfactory cortex (Narikiyo et al., 2018), we previously hypothesized that during the inhalation phase of respiration, external odor information is delivered to the higher cognitive areas in a feedforward direction from the lateral map of the olfactory bulb (OBl) via the superficial layer of the olfactory cortices (orange arrows in Figure 1; Mori and Sakano, 2011, 2021, 2022; 2024a). The higher cognitive areas then generate cognitive scene signals and transmit them back to the olfactory cortical areas during the late exhalation phase (purple arrows in Figure 1; Mori and Sakano, 2024b,2025a). However, it is yet to be studied which neural circuits coordinately regulate inhalation-correlated feedforward transmission of external odor information and exhalation-correlated top-down transmission of cognitive scene information.

Here, we propose that during the inhalation phase of respiration, basal forebrain (BF) cells suppress neurogliaform (NGF) cells in the superficial-layer. This suppression may disinhibit the pyramidal-cell dendrites in the superficial layer of the anterior piriform cortex (APC), resulting in the facilitation of feedforward transmission of sensory information via the superficial layer. In contrast, during the late exhalation phase, BF cells suppress the deep-layer NGF cells and disinhibit the pyramidal-cell dendrites in the same layer, resulting in the facilitation of top-down transmission of cognitive scene signals. We also propose that when the animals need to pay attention to the surrounding world, the medial prefrontal cortex (mPFC) sends command signals to the brainstem respiratory center to generate intentional inhalation and also to the BF cells to facilitate the feedforward transmission. If the animals need to pay attention to the cognitive inner world such as cognitive imagery and behavioral decisions, the mPFC commands the respiratory center to promote intentional exhalation and also directs the BF cells to facilitate the top-down transmission of cognitive scene information.

During feedforward transmission of input signals, the sensory stimuli are used for searching associative scene memory to evaluate the current situation. During the process of decision making, the top-down signals update the associative memory engram of sensory imagery through the same set of pyramidal cells as used for the recollection of the associated scene. It is assumed that repeated odor-object associative learning strengthens synaptic connections in both feedforward and top-down pathways, thus enhancing informational transduction in these pathways. In our previous perspective article (Mori and Sakano, 2025a), we described how the olfactory decision is made in the context of respiratory cycles: the inhalation phase is for identification of input sensory information and recollection of associative scene memory, and the exhalation phase is for valence decision and top-down directives of behavioral and emotional outputs. Based on this framework, here in this article, we will discuss respiration coordinated attentional switch from feedforward to top-down informational flow directed by the basal forebrain. We would like to propose that layer-specific blanket inhibition of pyramidal cells is responsible for this switching regulated by neurogliaform cells in the piriform cortex.

## Layer-specific blanket inhibition by neurogliaform cells

Figure 1A illustrates cortico-cortical pathways for the feedforward transmission of external odor information (orange solid arrows) and top-down transmission of cognitive scene signals (purple solid arrows) in the networks of the lateral olfactory cortical areas and higher cognitive areas. The lateral olfactory cortical areas include the lateral map of the olfactory bulb (OBl), lateral part of the anterior olfactory nucleus (AONl), anterior piriform cortex (APC), posterior piriform cortex (PPC), and cortical nucleus of amygdala (CoA) (Mori and Sakano, 2022, 2025a; b). The higher cognitive areas include the basal amygdala (BA), lateral entorhinal cortex (lEnt), lateral orbitofrontal cortex (ORBl), ventral agranular insular cortex (AIv), and posterior agranular insular cortex (AIp).

What are the neural circuit mechanisms for the respiration-coordinated feedforward and top-down transmission? Because of the slow, long-lasting, and non-selective nature of NGF-cell-evoked inhibition in pyramidal cells (Suzuki and Bekkers, 2010a; Overstreet-Wadiche and McBain, 2015; Suzuki et al., 2022), we speculate that NGF cells may play a key role in regulating the respiration-coordinated transmission. NGF cells are a type of inhibitory interneurons in the olfactory cortex, neocortex, hippocampus, and amygdala. They exert slow and long-lasting inhibition mediated by the GABA_*A*_ and GABA_*B*_ receptors in both pyramidal cells and inhibitory interneurons (Banks et al., 2000; Tamás et al., 2003; Szabadics et al., 2007; Oláh et al., 2009; Capogna, 2011; Capogna and Pearce, 2011; Chittajallu et al., 2013; Jiang et al., 2015). NGF cells have densely spaced presynaptic-terminals and release GABA in a target-independent cloud-like manner, causing slow and long-lasting inhibition of almost all dendrites located in the axon target layer (layer-specific blanket inhibition) (Overstreet-Wadiche and McBain, 2015; Jackson et al., 2018; Sakalar et al., 2022).

In the AONl, APC, and PPC, NGF cells are classified into two types; superficial-layer NGF cells (SL-NGF cells) and deep-layer NGF cells (DL-NGF cells) (Figure 1B; Suzuki and Bekkers, 2010b; Kay and Brunjes, 2014). SL-NGF cells are located in layer 1 and form dense axon terminals mostly in layer 1. This SL-NGF cell network indicates that SL-NGF cells may direct slow and long-lasting inhibition of all apical dendrites of semilunar cells (Se), superficial pyramidal cells (SP), and deep pyramidal cells (DP) in layer 1, i.e., blanket inhibition in the superficial layer (Figure 1B). In another word, the spike activity of SL-NGF cells may induce SL-specific blanket inhibition of all apical dendrites of principal cells. Because feedforward excitatory-synaptic inputs of olfactory sensory signals occur in these apical dendrites (Figure 1B, orange arrows with FF), blanket inhibition in SL would render principal cells irresponsive to the olfactory sensory inputs. Thus, we propose that SL-NGF cells may be inhibited during the inhalation phase so that the apical dendrites of principal cells are disinhibited (depolarized) and become ready to respond to the feedforward olfactory sensory-inputs during inhalation.

DL-NGF cells are located in layer 2 or 3 and distribute dense axon terminals in these layers. This DL-NGF cell network suggests that DL-NGF cells may provide slow and long-lasting inhibition of all deep dendrites of SP and DP in layers 2 and 3, i.e., blanket inhibition in the deep layers (Figure 1B). In another word, the spike activity of DL-NGF cells may cause DL-specific blanket inhibition of all deep dendrites in pyramidal cells. As the top-down excitatory synaptic-inputs of cognitive scene signals are generated in these deep dendrites (Figure 1B, a purple arrow with T-D), blanket inhibition in DL would render pyramidal cells irresponsive to the top-down cognitive scene-inputs. We therefore propose that DL-NGF cells may be inhibited during the late exhalation-phase so that all deep dendrites of pyramidal cells are disinhibited (depolarized) and become ready to respond to the top-down cognitive scene-inputs.

## Basal-forebrain neurons may induce respiration-coordinated inhibition of NGF cells

**FIGURE 2:**
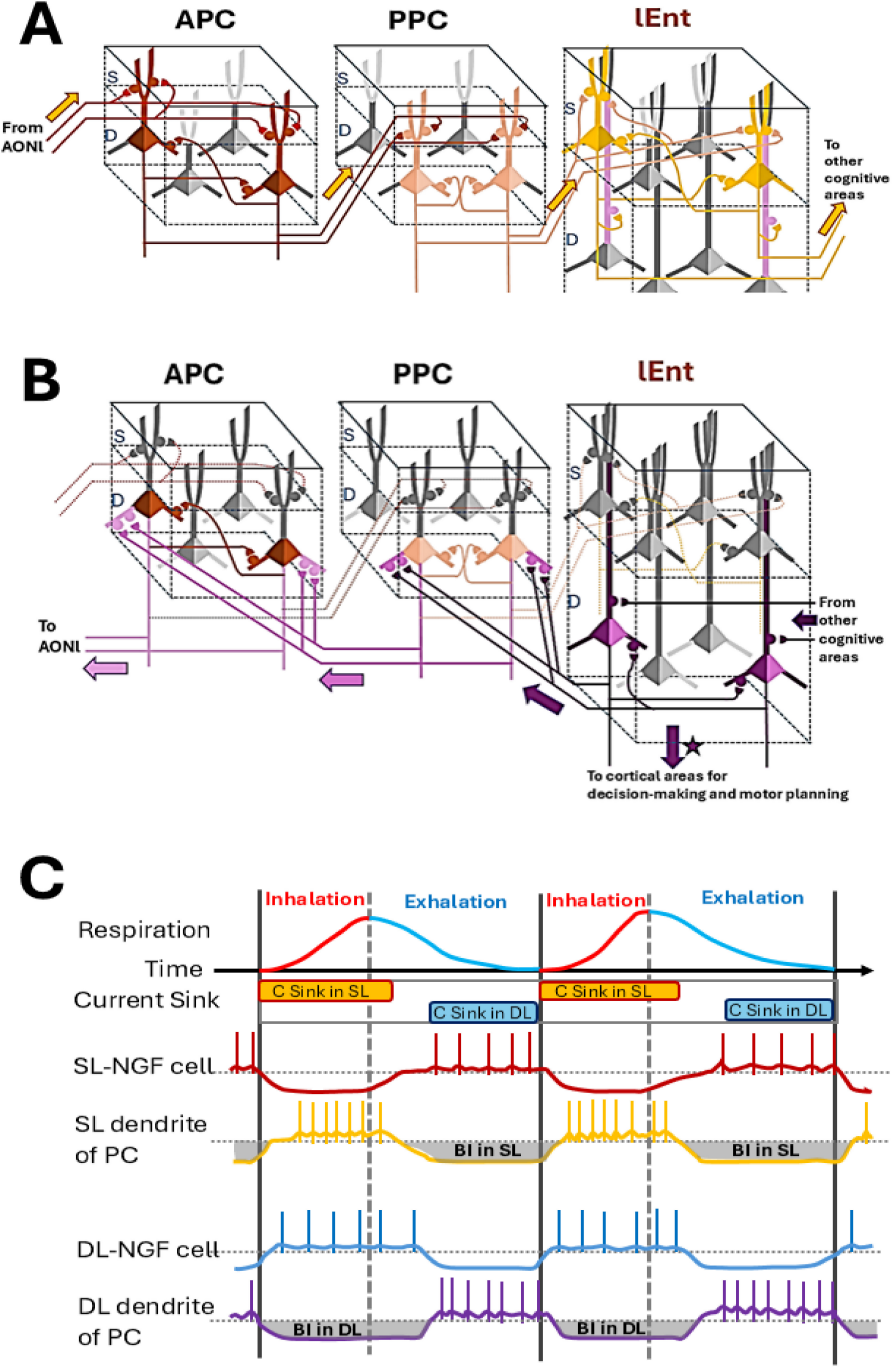
A laminar circuit and a functional model of layer-specific input gating in the olfactory cortex. **(A)** Feedforward transmission of odor signals in the network of APC → PPC → lEnt after the accumulation of odor-object associative memories. Odor signals of a specific object are transmitted from the AONl to the superficial layer of APC and activate memory engram cells for the object (pyramidal cells shown in dark red) in the APC. Other engram cells are shown in gray. The activated engram cells in the APC project axons to the superficial layer of the PPC and activate the PPC engram cells (pyramidal cells shown in pale brown). The activated engram cells in the PPC project their axons to the superficial layer of the lEnt and activate the engram cells in the superficial layer (layer 2/3 pyramidal cells shown in yellow). The engram cells in the lEnt receive converging sensory inputs from the visual, auditory, and somatosensory as well as the olfactory cortices and may represent the multisensory cognitive scene for the object. We postulate that after the accumulation of odor-object associative memory, odor-induced feedforward activation of engram cells in the APC and PPC activates the lEnt memory engram cells for the object. **(B)** Top-down transmission of cognitive scene signals in the network of lEnt → PPC → APC after the accumulation of odor-object associative memory. Engram cells in the deep layer of lEnt (layer 5 pyramidal cells shown in purple) generate cognitive scene signals for the object and transmit them to cortical areas for decision-making and motor planning (purple arrow with star) as well as to the deep layer of PPC. Top-down cognitive scene signals activate the engram cells in the PPC (pyramidal cells in pale brown). The activated PPC cells then activate the engram cells for the object in the APC via the top-down pathway. We postulate that activation of the engram cells in the lEnt results in the top-down activation of engram cells in the PPC and APC, resulting in the top-down recall of the olfactory object-imagery. **(C)** A model of membrane potential changes in NGF cells and principal cell dendrites in the olfactory cortex. During the inhalation phase, SL-NGF cells (magenta trace) demonstrate long-lasting hyperpolarization due to the inhibition by SL-projecting BF cells. Then, SL-dendrites of pyramidal cells (yellow trace) are depolarized (disinhibited) and become ready to respond to the feedforward (FF) odor inputs. During the late exhalation phase, DL-NGF cells (blue traces) show a long-lasting hyperpolarization due to the inhibition by DL-projecting BF cells. Then, DL-dendrites of pyramidal cells (purple trace) are depolarized and become ready to respond to the top-down inputs. Blanket inhibition of SL dendrites is shown in gray shadow with BI in SL. Blanket inhibition of DL dendrites is shown in gray shadow with BI in DL.

Which neuronal pathway, responsible for causing slow disinhibition (depolarization) of apical dendrites of principal cells, drives inhalation-correlated slow inhibition of SL-NGF cells? As extrinsic olfactory-sensory information is processed in phase with inhalation, the sensory signals may drive slow inhibition of SL-NGF cells and slow depolarization of apical dendrites. In the current-source-density analysis of local field potentials of the olfactory corticies, olfactory sensory input-activities are recorded by the gamma-oscillatory fast current sink in the SL, while slow depolarization of apical dendrites is monitored by the slow current sink in the SL (Figure 2C, orange bars with C Sink in SL) (Narikiyo et al., 2018). Ipsilateral naris-occlusion blocks inhalation-correlated olfactory sensory-inputs, and thus the gamma-oscillatory current sink in the SL of the olfactory cortex is shut off. However, the inhalation-correlated slow current sink in the SL occurs even under the naris occlusion (Narikiyo et al., 2018), indicating that the slow current sink is not driven by extrinsic olfactory sensory-inputs. We therefore assume that the intrinsic neural-circuit mechanism drives the slow current sink in the SL and slow depolarization of apical dendrites during inhalation. The current-source-density analysis also shows that naris occlusion does not reduce the slow current sink in the deep layer (Figure 2C, blue bars with C Sink in DL) that occurs during late exhalation. This observation indicates that the intrinsic neural-circuit mechanism drives the slow current sink in the DL and slow depolarization of deep dendrites during late exhalation. Then, which neural circuit drives slow depolarization of apical dendrites during inhalation, and which circuit drives slow depolarization of deep dendrites during late exhalation?

Basal forebrain neurons project to widespread cortical areas and play a key role in processing attentional and cognitive information in the cortex (Gaykema et al., 1990; Picciotto et al., 2012; Bloem et al., 2014; Gielow and Záborszky, 2017; Obermayer et al., 2017; Záborszky et al., 2018). Olfactory cortical areas receive the cholinergic and GABAergic inputs from the horizontal diagonal band (HDB) and magnocellular preoptic (MCPO) nuclei that are located in the rostral part of BF (Luskin and Price, 1982; Záborszky et al., 1986; Hook and Puche, 2023). It has been reported that a subset of HDB/MCPO neurons changes the firing rate selectively during cue-odor presentation (external attention period), while a different subset of HDB/MCPO neurons changes the activity specifically during the reward delivery period (internal attention period) in the odor-based go/no-go task (Nunez-Parra et al., 2020; Hanson et al., 2021). We therefore postulate that BF cells may be classified into two subsets of projection neurons: one (SL-projecting BF cells) projects axons to NGF cells in the superficial layer and selectively inhibits them during inhalation (external attention phase), and the other (DL-projecting BF cells) sends axons to NGF cells in the deep layer and selectively inhibits them during late exhalation (internal attention phase) (Figure 1B).

We speculate that SL-projecting GABAergic BF cells may show burst discharges and inhibit SL-NGF cells during inhalation, causing disinhibition of apical dendrites of pyramidal cells. Thus, burst discharges of SL-projecting GABAergic BF cells during inhalation may cause inhalation-phase-correlated facilitation of feedforward transmission of olfactory sensory information. We also speculate that SL-projecting cholinergic BF cells may fire just before the onset of inhalation and activate M1 muscarinic acetylcholine receptors on SL-NGF cells (Brombas et al., 2014), causing slow and long-lasting inhibition of SL-NGF cells during the inhalation phase. Thus, firings of SL-projecting cholinergic BF cells just before the onset of inhalation may facilitate feedforward transmission of olfactory sensory information during inhalation.

In the same token, DL-projecting GABAergic BF neurons may show burst discharges during late exhalation and inhibit DL-NGF cells, resulting in disinhibition of deep dendrites of pyramidal cells and facilitation of top-down transmission of cognitive scene information. DL-projecting cholinergic BF neurons may fire just before the late exhalation phase and inhibit DL-NGF cells for a long period during late exhalation, resulting in disinhibition of deep dendrites of pyramidal cells and facilitation of top-down transmission. In the neocortex, BF cholinergic inputs are powerful regulator of pyramidal cell synapses to somatostatin-expressing inhibitory interneurons (Urban-Ciecko et al., 2018). Therefore, other types of inhibitory neurons than NGF cells may also be involved in gating of deep-layer top-down input. It is also possible that the same or partially overlapping BF cholinergic inputs may generate different effects in the superficial and deep layers due to difference in postsynaptic nicotinic/muscarinic receptor profiles.

## The medial prefrontal cortex may coordinately regulate attentional switching and respiratory phase switching

The medial prefrontal cortex (mPFC) consisting of the dorsal anterior cingulate cortex, prelimbic cortex (PL), and infralimbic cortex (IL) mediates diverse cognitive functions, e.g., directing attention and making decisions (Euston et al., 2012; Porter and Sepulveda-Orengo, 2020; Zhang et al., 2020; Howland et al., 2022). The mPFC also plays a key role in controlling respiratory patterns and autonomic outputs (Vertes, 2006; Hassan et al., 2013; Bondarenko et al., 2014). As neuronal activity in the mPFC synchronizes with the respiratory cycle (Biskamp et al., 2017; Moberly et al., 2018; Basha et al., 2023), we speculate that the medial prefrontal cortex may generate the cortical command-signal for intentional inhalation and exhalation. We thus propose that the mPFC may regulate both attentional switching in the cortical networks and respiratory-phase switching in the respiratory central-pattern generator (CPG) in the brainstem (Smith et al., 2009; Krohn et al., 2023; Figure 1A). If the mammal needs to pay attention to the external world, the mPFC sends cortical command-signals to the brainstem respiratory CPG to initiate intentional inhalation and also to the BF cells to facilitate feedforward transmission of external sensory information. In this way, the mPFC may coordinate the attention to external sensory information with intentional inhalation. If the mammal needs to pay attention to cognitive imagery, behavioral decisions, and emotional valence in the inner world, then the mPFC sends command signals to the respiratory CPG to generate intentional exhalation and also to the BF cells to facilitate top-down transmission of cognitive scene information. In this way, the mPFC directs the attention to the cognitive inner world coordinately with intentional exhalation. It should be noted that respiration-phase-correlated switching of feedforward transmission to top-down transmission occurs only during wakefulness. These respiration-coordinated facilitation of informational transmission cannot be seen during the slow-wave sleep state (Manabe and Mori, 2013; Mori et al., 2013; Narikiyo et al., 2018).

## Feedforward operational state and top-down operational state

During wakefulness, there are two types of operational states in the network of lateral olfactory stream and higher cognitive areas (Figure 1A). One type occurs during intentional inhalation, which facilitates feedforward transmission of odor information and inhibits top-down transmission of multisensory cognitive-scene information. In this feedforward operational state, the network recalls memory of the multisensory cognitive imagery using the odor input pattern as a search tag (Mori and Sakano, 2025a). In other words, the brain directs the working mode to the feedforward operational state to attend to the external odor information. This recalls memory of the cognitive object-imagery, which is encoded in the feedforward connectivity to higher cognitive areas.

Another type of operational state occurs during intentional exhalation, facilitating top-down transmission of cognitive object-imagery information and inhibiting feedforward transmission of odor information. In this top-down operational state, the network recalls memory of the olfactory imagery based on the multisensory cognitive imagery of the object (Mori and Sakano, 2025a). In other words, the brain switches the working mode to the top-down operational state in order to attend to the internal cognitive-scene information. This recalls olfactory object-imagery memory, which is encoded in the top-down connectivity from the higher cognitive areas to the olfactory cortices. We speculate that if the brain needs to attend to the external odor information, it instructs the respiratory CPG to elicit intentional inhalation and commands the BF circuits to switch to the feedforward operational state. If the brain needs to attend to the internal cognitive-scene information, it instructs the CPG to elicit intentional exhalation and commands the BF circuits to switch to the top-down operational state. It is interesting that the same set of pyramidal cells and their recurrent collateral excitatory-synapses are used for both feedforward transmission and top-down transmission under the inhibitory control of NGF cells. One may wonder how this single-track train operation is regulated without head-on collision. As we proposed in the present study, it appears that respiration-coordinated layer-specific blanket inhibition is responsible for avoiding this confusion in the sensory cortices.

We further speculate that the synchronization of the respiratory phase and operational state occurs not only in the network of the olfactory cortex and its higher cognitive areas, but all across the sensory networks of the visual/auditory/somatosensory cortices and their higher cognitive areas. If the brain needs to attend to external sensory information, it instructs intentional inhalation and switches to the operation mode for feedforward processing of all sensory information. If the brain needs to attend to multisensory cognitive scenes, it instructs intentional exhalation and switches to the operation mode for top-down processing of cognitive scene information. To cognize and react to the changes in the external world, the brain needs to flexibly and intentionally switch its attention to either the surrounding external world or cognitive internal world. We propose that the network of “mPFC → BF → all sensory cortices” plays a key role in coordinately regulating both attentional switching and respiratory-phase switching.

In the human brain, respiration-correlated switching of external attention and internal attention is prominent during language communication. After establishing associative memory of spoken words (such as “apple”) with the cognitive object-imagery, humans communicate with each other using learned language. In the listening phase, the human brain attends to the auditory inputs of spoken words (external attention) and facilitates feedforward transmission of auditory information to higher cognitive areas via the auditory cortices. During active hearing, humans show either inhalation or breath holding, but not active exhalation. Feedforward transmission of auditory information recalls associative memory of the cognitive object-imagery. Once higher cognitive areas generate the cognitive object-imagery, the brain switches its attention to the inner world, changes the respiratory phase to exhalation, and sends the top-down signals of cognitive object-imagery to each sensory cortex.

Transmission of the cognitive object-imagery to the visual cortex may recall the memory engram of visual object-imagery (for example, “round shape and red color” of apple), while the transmission to the olfactory cortex may recall the olfactory object-imagery (“fruity smell” of apple). Therefore, the human brain may require coordinated switching from feedforward transmission of auditory information to top-down transmission of the cognitive object-imagery in order to comprehend the integrated imagery of object (“red, round, and fruity” apple) based on auditory inputs of spoken words.

## Differential usage of memory engram cells in the feedforward and top-down transmission

Our respiration-phase selective model suggests that separate neuronal populations in the BF may regulate the layer-specific input-gating in the olfactory cortex through inhibitory interneurons. Then, How does the inhalation-phase-selective odor-input utilize memory engram cells in the olfactory cortex and higher cognitive areas in generating the cognitive object-imagery? Also, How does the exhalation-phase selective top-down cognitive scene input utilize these engram cells in generating the olfactory object-imagery? Figure 2A illustrates feedforward transmission of odor signals from a specific object in the network of APC → PPC → lEnt after the accumulation of associative memory of the odor information with the multisensory cognitive imagery of the object (Mori and Sakano, 2025a). During the inhalation phase, odor signals of a specific object are delivered into the superficial layer of APC via the OBl and AONl, and activate a particular set of memory engram cells for the object in the APC (pyramidal cells shown in dark red). The engram cells are connected to each other via recurrent excitatory synapses and represent the olfactory imagery of an object in the APC. APC engram cells representing the object project their axons to the superficial layer of PPC and may drive engram cells in the PPC (pyramidal cells shown in pale brown). The activated PPC engram cells project their axons to the engram cells for the object in the superficial layer of lEnt (layer 2/3 pyramidal cells shown in yellow).

Engram cells for the object in the lEnt receive converging sensory inputs from the olfactory, visual, auditory, and somatosensory cortices and may represent the multisensory cognitive imagery of an object (Burwell, 2000; Igarashi et al., 2014). We postulate that odor-induced activation of engram cells in the APC and PPC results in the feedforward activation of the engram cells in lEnt (Figure 2A). In another word, olfactory sensory input uses the preferential feedforward synaptic-connections (APC → PPC → lEnt) of engram cells (Ryan et al., 2015) in generating the cognitive object-imagery. The feedforward synaptic connections of memory engram cells may convert the input olfactory pattern as a QR code into the polymodal cognitive imagery of an object.

Figure 2B illustrates top-down transmission of cognitive signals of the object imagery in the network of lEnt → PPC → APC after the accumulation of associative memory of odor information with the cognitive object imagery. Engram cells for a specific object in the deep layer of the lEnt (layer 5 pyramidal cells shown in purple) generate cognitive scene signals during the late exhalation phase and transmit them to the cortical areas for decision-making and motor planning (a purple arrow with a star) as well as to the deep layer of PPC. Top-down cognitive scene signals activate the same set of engram cells in PPC as those activated during the feedforward transmission of odor information (pyramidal cells shown in pale brown) (Mori and Sakano, 2025a). Activated engram cells transmit cognitive scene signals to the engram cells in APC (shown in dark red). We postulate that after the establishment of associative learning of odors with objects, internal activation of engram cells of an object in the deep layer of lEnt results in the top-down activation of engram cells in the PPC and APC. In another word, top-down cognitive scene input uses the preferential top-down synaptic connections (lEnt → PPC → APC) of engram cells in generating the olfactory object-imagery. The top-down synaptic connections of engram cells may convert abstract cognitive object-imagery into the concrete olfactory object-imagery.

In the off-line state such as non-REM sleep and awake resting with internal attention, the feedforward transmission of external sensory information is suppressed in the sensory cortices and engram cells in the higher cognitive areas cannot be activated by sensory input. However, the preferential top-down synaptic connections of memory engram cells can repeatedly be activated without external sensory inputs during the off-line state (Buzsáki, 2015). For the odor-based conscious object-cognition, the usage of feedforward connections of engram cells may be needed during inhalation, while the usage of top-down connections may be needed during late exhalation.

## Discussion

It is generally thought that repeated associative learning enforces synapse formation and strengthens existing neural connections (Poo et al., 2016; Bocchio et al., 2017). This activity-dependent synaptic plasticity appears to be responsible for establishing and reorganizing the memory engrams and neural networks. Associative memory of a specific sensory input with the multisensory cognitive object-imagery is generated based on activity-induced synapse potentiation in both the feedforward sensory pathway and top-down cognitive signal pathway. Linking a specific language-word to the cognitive imagery is established by such synaptic potentiation in the feedforward pathway. Updating the memory engram by top-down cognitive information is another example of activity-dependent synapse enforcement. Although this Hebbian rule (Hebb, 1949) is well-accepted by neuroscientists, we still need to clarify the question of how the differential usage of memory engram cells (feedforward and top-down) is regulated at the molecular level in organizing, generating, and renewing memory engrams.

It has been reported that protein synthesis is needed for fixing the updated memory engrams (Nadar et al., 2000; Ryan et al., 2015). This process probably requires reinforcement of synapses in a neural-activity dependent manner. The Sema7A/PlexnC1 system is reported to be involved in the imprinted memory formation of the mouse olfactory system (Inoue et al., 2018, 2021). These signaling molecules mediate synapse formation within the glomeruli in an activity-dependent manner during the critical period. The same or similar signaling systems may also be utilized for synapse formation in rewriting the memory engrams. As Sema7A is highly expressed in the CA2 region of the hippocampus, it will be interesting to analyze the conditional knock-out (KO) mouse of Sema7A for CA2.

Consciousness is the state of being aware of external objects in the environment or internal subjects including ourselves. Changes in the outer world are detected by various sensory systems. We also pay attention to the inner world by detecting mental and physical situations, and evaluate the status of ourselves by recalling the past, while considering the present, and predicting the future. It is intriguing that the respiratory cycle is closely correlated with attentional switching between the surrounding outer world and cognitive inner worlds. As already mentioned earlier, detection and identification of incoming sensory information take place during the inhalation phase of respiration, directing attention to the surrounding world. In contrast, decision making for output behaviors and emotions takes place during the following exhalation phase. In this perspective article, we discussed how these attentional switching and respiratory-phase switching are coordinately regulated at the neural circuit level.

Attention is defined as the concentration of awareness directed toward a particular object excluding other objects. Attention is triggered by the sensory input from the surrounding world that activates the engram of associative memory, causing recollection of the previous memory-scene. Once attention is focused on a specific object of interest, we try to find what it is (identification), determine whether it is good or not (evaluation), and collect further information (investigation). Attention is also directed toward a particular subject in the imaginary world, which is raised by spontaneous firing of memory engrams when we are relaxed, causing us to wondering whether the current situation is satisfactory or not. The cognitive brain areas make output decisions by referring to the memory scene in the imaginary world, and direct both behavioral and emotional responses. As such attentional switching is closely coordinated with switching of respiratory phases, we proposed that both types of switching may be intentionally regulated by the medial prefrontal cortex (Mori and Sakano, 2024a,2025a; b).

Attention is not just a kind of consciousness during wakefulness. We are not just looking at the passing-by scene in the surrounding world or dreaming a daydream without taking behavioral actions or moving our mind. After paying attention to a specific object, we evaluate the situation and purposefully take actions. Then, What makes us motivated to respond to a situation? How is the valence decision made, and what kind of neuromodulators are involved in generating behavioral and emotional outputs? Such decision is made based on the two kinds of memories. One is individuals’ memory that is stored in the memory engrams for learned decisions. The other is species’ memory that is encoded in DNA as a result of natural selection during evolution for innate decisions. It is important to study how the brain makes a measurement of the distance between the current situation and what it should ideally be. We humans may be applying the self-localization capability (Mao and Gu, 2025) to the self-positioning in the valence space of the imaginary world.

Self-cognition arises when we (first-person self) find ourselves as an object (third-person self) in the surrounding world. Self-awareness occurs when we recognize ourselves in the cognitive world. Attention to ourselves initiates thinking whether the surrounding situation and our current status are satisfactory. Once the present self is recognized and evaluated in reference to the ideal status, we are motivated to take actions by formulating a strategy that aims to the goal or improve the situation. As we discussed, respiratory cycle is closely coordinated with thinking about ourselves, evaluating the situation, becoming motivated, and taking actions.

Animals intentionally control respiratory cycles, when they are actively thinking and moving around. However, when they are faced with danger and crises, they occasionally keep quiet and hold their breaths without moving. It has been reported that predators’ scents, e.g., the fox odor trimethyl-thiazoline (TMT), induce strong fear responses inducing avoidance and freezing (Kobayakawa et al., 2007). There are about 20 odorant receptors (ORs) responsive to TMT. One of the TMT-reactive ORs, Olfr1019, when photo-activated alone, induces freezing but not avoidance (Saito et al., 2017). Increase in the stress hormone ACTH is not observed during freezing when the knock in (KI) mouse of the *olfr1019* linking to the channel-rhodopsin gene is photo-illuminated. The KO mice of the *olfr1019* fail to show freezing, however, normally demonstrate avoidance toward TMT. Therefore, freezing is not a stress-induced response, but a separate behavioral decision of “immobility.” In this freezing, the photo-illuminated *olfr1019* KI mouse decides not to move and waits for the situation to change, resetting the strategy for survival. During this immobility, which area in the brain is responsible for blocking the output behaviors and emotional movements to keep quiet? We humans also experience “holding our breaths” when we encounter crisis, danger, or fear. It will be interesting to determine whether the same brain region used for the attentional switch, is responsible for directing such breath holding reactions in immobility.

During deep sleep, we do not think or take actions. However, when we are having dream, we partly awake and pay attention to the dreaming scene. What is happening to our consciousness in the dream? Which part of the brain that processes the sensory signals is blocked, and which part is still functioning? Such issues of consciousness need to be studied also in relation to the sleeping mechanism. Anesthesia paralyzes our sensory perception and output responses at various stages (Garcia et al., 2010). It will be important to determine which step of attentional responses is targeted by a specific anesthetizing agent. These studies on sensory processing, learning, and attentional switching by the neuroscience will give new insight into our understanding of consciousness, thinking, motivation, and decision making that used to be studied by the humanities and social sciences.

## Data Availability

The original contributions presented in this study are included in this article/supplementary material, further inquiries can be directed to the corresponding authors.
